# Leukemia cutis presenting as hyperpigmented patches. A rare presentation

**DOI:** 10.1002/ccr3.6386

**Published:** 2022-10-03

**Authors:** Nastaran Namazi, Zahra Asadi Kani, Ali Kaddah, Reem Diab

**Affiliations:** ^1^ Skin Research Center Shahid Beheshti University of Medical Sciences Tehran Iran; ^2^ Department of Surgery, Shohada‐e Tajrish Hospital Shahid Beheshti University of Medical Sciences Tehran Iran

**Keywords:** hyperpigmentation, leukemia, leukemia cutis

## Abstract

Leukemia cutis is a cutaneous manifestation of leukemia. Herein, we present a rare cutaneous manifestation of leukemia cutis in a patient with myeloid leukemia m5, characterized by hyperpigmented red‐to‐brown patches on face and upper trunk. To our knowledge, hyperpigmented patches secondary to leukemia cutis is rarely described in the literature.

## Graphical abstract

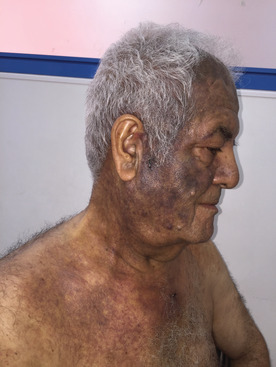

## 1 INTRODUCTION

Leukemia is a potentially fatal and life‐threatening neoplasm affecting the hematopoietic system.^1^ Direct invasion of leukemic neoplastic cells in the skin layers is named leukemia cutis.^1^ Leukemia cutis is seen in various frequencies in leukemia subtypes, but it is most commonly seen in acute myelomonocytic leukemia (AML‐4) and AML‐M5, which have the highest rates of skin manifestations.^2^ Approximately, about 2.9%–3.7% of AML cases present with leukemia cutis.^2^

We report a case of a 76‐year‐old man with skin rash who was diagnosed later with acute myeloid leukemia (AML). The patient presented with hyperpigmented patches on the face and upper trunk with ocular and oral mucosal changes upon examination. Further workup revealed patient having acute myeloid leukemia (AML‐M5), and skin biopsy showed infiltration with myeloblasts.

## 2 CASE PRESENTATION

**FIGURE 1:**
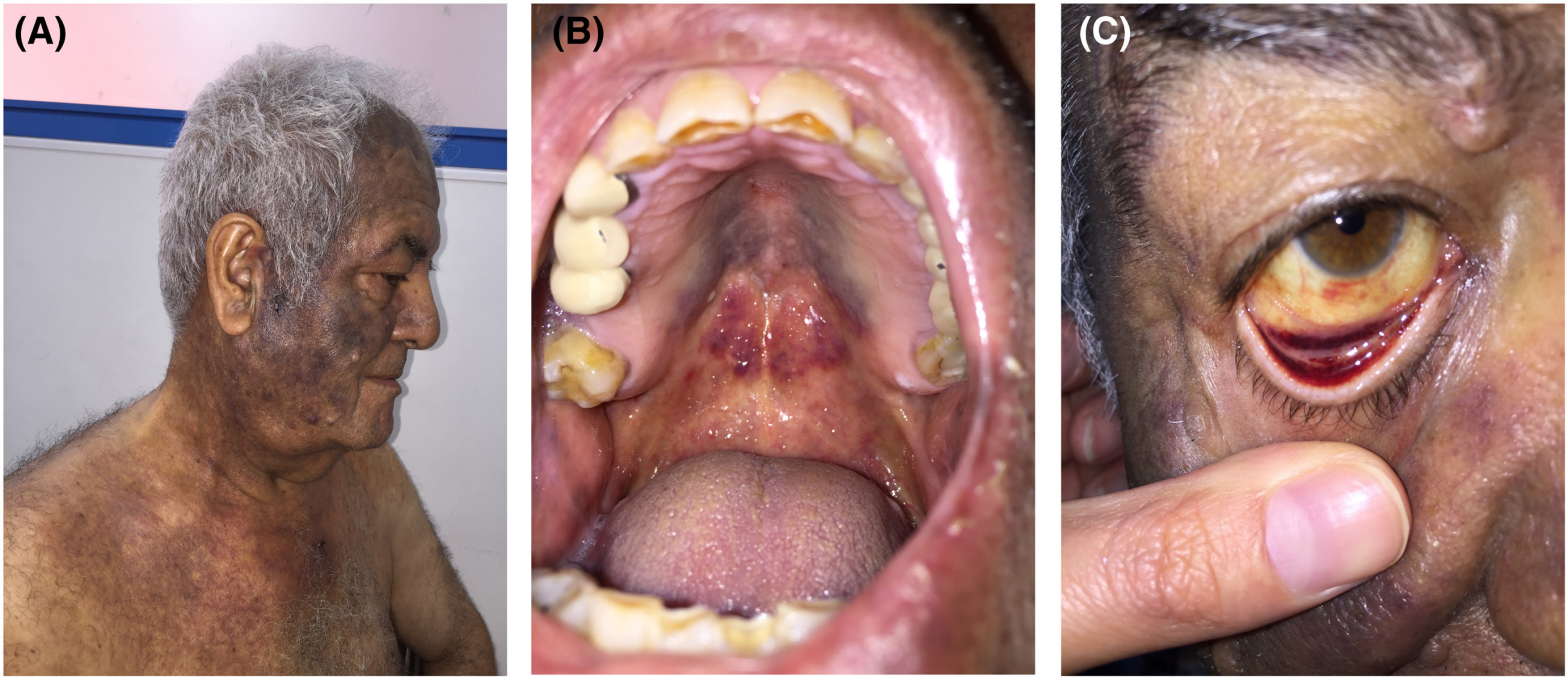
(A) hyperpigmented patches on face and upper trunk. (B) violaceous plaques on the upper palate. (C) yellow sclera with red to purple patches on the inner aspect of lower eyelid.

A 76‐year‐old man with no significant past medical history presented to our clinic with a 6 months skin hyperpigmentation and fatigue. The patient was treated with skin‐lightening and antisolar creams but with no improvement. Clinical examination showed brown to violet hyperpigmented patches on face and upper trunk. Mucosal examination showed violaceous plaques on the upper palate in addition to yellow sclera with red to purple patches on the inner aspect of both lower eyelids (Figure 1). Physical examination showed lymphadenopathy especially in axillary area. Laboratory evaluations revealed white blood cell 4.6 × 10^3^/L with neutrophil and lymphocyte differentiation (18% and 50%, respectively), hemoglobin 10.5 g/dl, and platelet count of 72 × 10^3^ μl.

**FIGURE 2:**
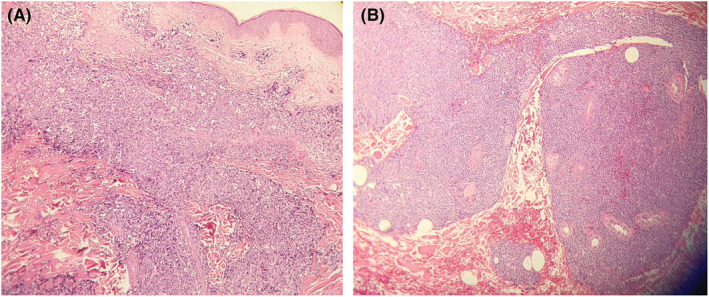
Dense perivascular and periadnexalnodular infiltrate of atypical mononuclear cells in dermis.

Two skin biopsies from face and chest were taken with differential diagnoses of angiosarcoma, lymphoma, and lichen planus. The result showed dense dermal multinodular perivascular and periadnexal infiltration with atypical mononucleated cells with IHC evidence consistent with leukemic dermal infiltration (Figure 2). Immunohistochemistry (IHC) staining was carried out according to morphological findings and showed infiltrated cells with irregular indented nuclei and high nuclear/cytoplasmic ratio, which was positive for leukocyte common antigen (LCA). No CD20, CD79a, CD3, CD45, CD30, MPO, or CD68 were detected in infiltrated cells. Clinical and histopathological findings in addition to the flow cytometric immunophenotyping of peripheral blood were compatible with myeloid leukemia m5.

## 3 DISCUSSION

Patients with underlying hematologic malignancies can present with a diversity of non‐specific reactive skin lesions or specific infiltrates.^3^ Leukemia cutis is a cutaneous infiltration of neoplastic leukocytes, which has multiple clinical presentations including erythematous papules, nodules, plaques, bullae, noduloulcerative lesions, and even erythroderma.^3^ The lesions are often firm or/and hemorrhagic lesions. The most common locations are face, head, neck, and trunk.^3^

In addition to usual typical skin manifestations, the recorded unusual manifestations of leukemia cutis were summarized in retrospective study in 2021 by Yung‐WeiChang, which included leonine faces, figurate cutaneous lesions, fingertip hypertrophy, erythema nodosum, guttate psoriasis, chronic paronychia, leukemic vasculitis, and Sister Mary Joseph's nodule.^4^

Our patient was complaining of hyperpigmented patches on the face and upper trunk from 6 months before presenting to our clinic, and unfortunately, the patches were treated as skin pigmentation.

Involvement of skin can be seen in any leukemia subtype, but most commonly seen in acute myeloid leukemia (AML) with monocytic or myelomonocytic and chronic lymphocytic leukemia (CLL), and rarely in chronic myeloid leukemia (CML), which can be indicative for blast transformation.^5^

The diagnosis of leukemia cutis is usually achieved by histological study and immunophenotyping.^6^ Histological findings vary according to the type of leukemia and most commonly present as perivascular and periadnexal dermal infiltrate.^6^, ^7^ Additional occasional finding includes stromal fibrosis, ulceration, dermal vessel thrombus, and subcutaneous.^7^

Currently, there is no specific treatment for leukemia cutis, and cutaneous eruption usually resolves following treatment of underlying leukemia.^8^

To our knowledge, hyperpigmented patches secondary to leukemia cutis are rarely described in the literature. Dermatologists could have an important role in diagnosis of internal diseases including leukemia. Postponing the diagnosis of leukemia cutis means delaying the diagnosis of the underlying leukemia, which lead to poor prognosis. Early diagnosis of leukemia cutis can lead to better outcomes and may direct to an underlying malignancy.

### AUTHOR CONTRIBUTORS

NN was involved in the diagnosis and management of the patients and has been responsible for the clinical part of the manuscript. ZAK reported the result of histopathological evaluation. NN, RD, and AK did literature review and drafted the manuscript. NN was responsible for final editing of the manuscript and coordinated the study. All authors have read and approved the final manuscript.

## FUNDING INFORMATION

This research did not receive any specific grant from funding agencies in the public, commercial, or not‐for‐profit sectors.

## CONFLICT OF INTEREST

The authors have no conflicts of interest to declare.

## CONSENT

Written informed consent was obtained from the patient for publication of this case report and accompanying images.

## Data Availability

Data openly available in a public repository that issues datasets with DOIs
